# Supplementary material for: The interactome of intact mitochondria by cross-linking mass spectrometry provides evidence for coexisting respiratory supercomplexes
Source: Mol Cell Proteomics. 2017 Dec 8;17(2):216–32. doi: 10.1074/mcp.RA117.000470 (PMC5795388; doi:10.1074/mcp.RA117.000470)
Supplement: Supplemental Data [file supp_RA117.000470_133922_0_supp_23978_fzffwf.zip › spectra_annotation/mito_DR_spectra_annotation/308-1-4-1-22-1.pdf]

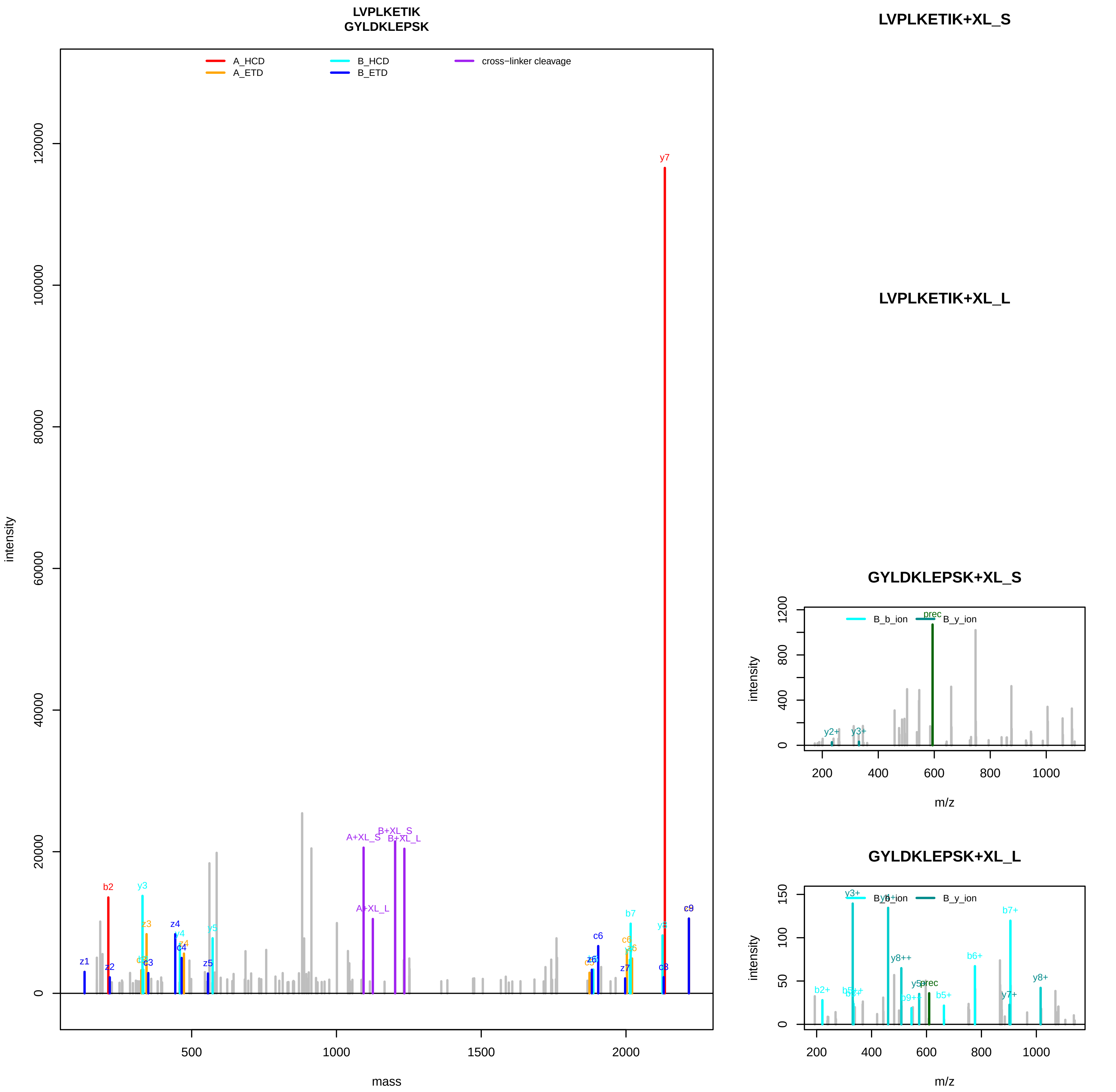

LVPLKETIK
GYLDKLEPSK
A_HCD
A_ETD
B_HCD
B_ETD
cross−linker cleavage
intensity
mass
0
20000
40000
60000
80000
100000
120000
500
1000
1500
2000
y7
b2
y3
z3
z4
y4
c4
z1
z2
c3
z5
y5
A+XL_S
B+XL_S
B+XL_L
A+XL_L
c6
z6
c6
y6
b7
z7
c8
c9
LVPLKETIK+XL_S
LVPLKETIK+XL_L
GYLDKLEPSK+XL_S
B_b_ion
B_y_ion
prec
y2+
y3+
m/z
0
400
800
1200
200
400
600
800
1000
GYLDKLEPSK+XL_L
y3+
y8++
b6+
b7+
y8+
b2+
b5+
y5+
prec
y7+
0
50
100
150
